# Trends in adherence to recommended physical activity and its association with cardiovascular risk factors in US adults with cardiovascular disease: a cross-sectional study

**DOI:** 10.1186/s12872-022-02854-9

**Published:** 2022-09-16

**Authors:** Yuhong Cheng, Lin Mou, Zhiliang Li

**Affiliations:** Department of Neurosurgery, Linfen Central Hospital, Linfen, 031412 Shanxi China

**Keywords:** Physical activity, Cardiovascular disease, Stroke, Risk factors

## Abstract

**Background:**

Being physically active is important for cardiovascular health. This study aimed to examine the trend in adherence to the physical activity guidelines (PAG) for aerobic activity among US adults with a history of cardiovascular disease (CVD) and evaluated its association with cardiovascular risk factors.

**Methods:**

We studied participants from the national health and nutrition examination survey 2007–08 to 2017–18. Regression models were used to evaluate the significance of the trend and the association between adherence to the PAG with cardiovascular risk factors.

**Results:**

A total of 3638 participants were reported to have a history of CVD. The proportion of adherence to PAG significantly increased from 41.5% in 2007–08 to 54.3% in 2017–18. Males had a higher proportion of adherence compared to the females, while the trend in adherence was only significant in females. Adherence to the PAG was significantly associated with decreased levels of waist circumference, body mass index, hemoglobin A1c, and triglycerides.

**Conclusions:**

There is a significant increase in the proportion of adherence to the PAG among US adults with a history of CVD from 2007–08 to 2017–18, and adherence to the PAG was associated with improvement in cardiovascular risk factors.

## Introduction

Regular physical activity is important to improve health. Inadequate physical activity is a worldwide public health problem, accounting for approximately 6–10% of major non-communicable diseases and 9% of premature mortality [1]. The lack of physical activity is linked to nearly $24.7 billion in annual health care costs in the United States [2]. The Physical Activity Guidelines for Americans (PAG) released by the US Department of Health and Human Services recommended that adults obtain at least 150 min/week of moderate intensity physical activity, 75 min/week of vigorous intensity physical activity, or a combination of both [3, 4]. However, according to a recent report, the proportion of US adults adhering to the PAG for aerobic activity has not increased from 2007–08 to 2015–16, while sedentary time increased significantly during this period [5].

Cardiovascular disease (CVD) is the leading cause of death in the US. According to the data from National Health and Nutrition Examination Survey (NHANES) 2015–2018, the prevalence of CVD (comprising chronic heart disease, heart failure, stroke, and hypertension) in adults aged 20 years or older is 49.2% overall. The CVD prevalence excluding hypertension is 9.3% overall (26.1 million in 2018) [6]. Adults who are physically active have a reduced risk of CVD and cardiovascular mortality than those who do not [4, 7]. Nonetheless, the proportion of adults who met the PAG for aerobic activity is low [8]. There is no report about the changes in adherence to the PAG over time in the US population with CVD. The association of adherence to the PAG with the cardiovascular risk factors among adults with a history of CVD has not been evaluated previously.

In this study, we analyzed the data from NHANES to report the trends in adherence to the PAG for aerobic activity among US adults with CVD and evaluated its association with cardiovascular risk factors.

## Methods

The NHANES is a large, nationally representative cross-sectional survey conducted in 2 years cycles in the US. It focuses on health conditions and behaviors, physical examination findings, and laboratory results. The NHANES data are collected by the Centers for Disease Control and Prevention National Center for Statistics with a multistage, probability cluster survey method. The study protocols were approved by the National Center for Health Statistics Research Ethics Review Board and written informed consent was obtained from participants. We used data from 6 consecutive 2 year cycles spanning 2007–08 to 2017–18 because the NHANES used a different questionnaire to assess physical activity before 2007–08. We limited our participants to non-pregnant adults (aged ≥ 18 years) with available information on their history of CVD and physical activity.

Demographic and health-related information was collected by standardized questionnaires. Race/ethnicity was self-reported and categorized as Mexican American, non-Hispanic white, non-Hispanic black, and others. Educational level was assessed by asking the highest level of school completed or the highest degree received and classified as less than high school, high school graduate, some college, and college graduate or higher. The income was measured by the income-to-poverty ratio, which was defined as annual family income divided by the poverty threshold adjusted for family size and inflation. Current smoking and alcohol use were based on questionnaires about whether participants were currently smoking and drinking or not.

History of CVD was ascertained by the self-report, and CVD included stroke, congestive heart failure, angina, and myocardial infarction in the current study.

Body mass index (BMI) was calculated as weight in kilograms divided by the height in meters squared, both measured during the standardized examination. Obesity was defined as BMI of 30 or greater. Waist circumference was measured to the nearest 0.1 cm at the superior border of the iliac crest. Blood pressure (BP) was assessed using 3 consecutive standardized blood pressure readings during the physical examination. Hypertension was defined as systolic BP ≥ 130 mm Hg, diastolic BP ≥ 80 mm Hg, or the use of antihypertensive medications. Blood samples were collected during the examination, stored at − 20 °C, and sent to the central laboratories for the measurement of hemoglobin A1c, total cholesterol, high-density lipoprotein-cholesterol (HDL-C), and triglycerides. Diabetes was defined as hemoglobin A1c ≥ 6.5% or the use of antidiabetic medications, and dyslipidemia as total cholesterol ≥ 240 mg/dL or the use of lipid-lowering medications.

Physical activity was evaluated by the Global Physical Activity Questionnaire, which measured leisure-time, work-related, and transportation-related physical activity. The questions evaluating leisure-time and work-related physical activity included the intensity (moderate versus vigorous), frequency (per week), and duration (minutes) in a typical week. The transportation-related physical activity was evaluated by asking the number of days in a typical week and the mean duration per day that participated in the activity. The NHANES suggested a metabolic equivalent score of 4.0 for transportation-related physical activity, 4.0 for moderate intensity activity, and 8.0 for vigorous intensity activity. In this way, the transportation-related activity was counted as moderate intensity activity. The total amount of physical activity was calculated as minutes of moderate intensity activity plus twice the minutes of vigorous intensity activity of all 3 domains [9]. According to the guidelines, adherence to the PAG for aerobic activity was defined as participating in at least 150 min per week of moderate intensity activity [4].

### Statistical analysis

According to the recommendation from NHANES, appropriate 12 year sampling weights were constructed to make sure the results accurately estimate associations and variances [10]. Statistical analysis was performed in R version 4.1.0 with the “Survey” package after accounting for the complex sampling design. All statistical tests were 2-sided, and *P* < 0.05 was considered statistically significant. Continuous variables were expressed as means [standard error or 95% confidence interval (CI)] and categorical variables as percentages (standard error).

The logistic regression model was used to test the trend in adherence to the PAG for aerobic activity across time, with the survey cycle as an independent variable. We first test the nonlinearity of the trend by adding a quadratic term of the survey cycle into the regression model. If it was insignificant, we then tested the linearity of the trend. We further tested the significance of the trend by adjusting covariates in the regression models. The following variables included age, gender, race/ethnicity, education, income, smoke, alcohol use, obesity, diabetes, hypertension, and dyslipidemia. To test whether the trend differed across different subgroups by age (18–44 years, 45–64 years, and 65 years or older), gender, race/ethnicity, education, and income, a two-way interaction term between the survey cycle and subgroup status was added into the model. We also performed subgroup analysis by diagnosis. There were three different diagnoses including patients with stroke alone, with heart disease alone, and with both stroke and heart disease.

The linear regression model was used to examine the association between adherence to the PAG with different cardiovascular risk factors. We first tested the association without adjusting for the aforementioned covariates, and then we tested the association with the adjustment. The results were presented as standardized beta coefficients.

## Results

The baseline characteristics of the participants are presented in Table 1. We included 3638 participants 18 years or older from NHANES 2007–08 to 2017–18. No significant changes in the mean age, gender distribution, income-to-poverty ratio, current smoke rate, waist circumference, the proportion of participants with a history of hypertension and diabetes, and the level of hemoglobin A1c and HDL-C were observed over time.

**Table 1:** Characteristics of participants with CVD, NHANES 2007–08 to 2017–18

	2007–08	2009–10	2011–12	2013–14	2015–16	2017–18	*P* for trend
Numbers	671	645	544	568	583	627	
Mean age, years	65.0 (0.6)	64.5 (0.6)	64.3 (0.8)	65.3 (0.9)	65.3 (0.8)	65.3 (0.6)	0.384
Male, %	52.5 (2.6)	57.8 (2.3)	52.2 (2.2)	52.6 (3.4)	54.9 (2.5)	57.0 (2.9)	0.486
*Race/ethnicity, %*							
Mexican American	7.1 (1.7)	8.1 (2.7)	9.7 (2.4)	8.5 (1.8)	9.1 (1.7)	8.3 (1.6)	0.658
Non-Hispanic White	74.4 (3.7)	75.2 (3.7)	70.8 (3.5)	75.2 (2.9)	67.1 (3.6)	71.1 (3.4)	0.240
Non-Hispanic Black	12.7 (2.1)	12.1 (1.7)	12.7 (2.9)	11.1 (1.4)	13.0 (2.5)	11.0 (1.9)	0.659
Other	5.7 (1.2)	4.6 (1.2)	6.8 (1.5)	5.3 (1.0)	10.8 (2.0)	9.6 (2.3)	**0.015**
*Education, %*							
Less than high school	31.0 (2.7)	28.6 (2.4)	26.3 (3.3)	22.1 (2.4)	19.2 (2.1)	15.8 (2.9)	** < 0.001**
High school graduate	28.3 (2.5)	26.7 (2.2)	27.0 (3.8)	26.9 (1.7)	25.1 (2.3)	31.5 (2.7)	0.531
Some college	23.3 (3.1)	26.3 (2.1)	28.4 (3.1)	29.6 (1.5)	36.2 (3.0)	28.7 (2.6)	**0.023**
College graduate	17.3 (2.4)	18.4 (2.4)	18.3 (2.3)	21.3 (2.5)	19.4 (2.7)	23.9 (3.8)	0.120
IPR	2.5 (0.1)	2.8 (0.1)	2.5 (0.1)	2.4 (0.1)	2.5 (0.1)	2.8 (0.1)	0.496
IPR < 1.3 (%)	29.0 (2.8)	25.1 (2.4)	31.6 (2.8)	31.7 (2.9)	30.5 (2.8)	23.3 (1.9)	0.040*
Current smoke, %	20.1 (15)	19.5 (2.1)	23.6 (2.8)	22.0 (2.0)	26.2 (3.8)	18.4 (2.3)	0.707
Current alcohol use, %	67.4 (2.7)	70.0 (2.5)	74.6 (2.5)	73.3 (2.1)	71.4 (3.5)	65.9 (2.9)	0.006*
BMI	30.0 (0.3)	30.9 (0.4)	30.1 (0.4)	30.6 (0.5)	31.2 (0.6)	31.1 (0.3)	**0.017**
Obesity, %	41.6 (2.1)	49.0 (1.6)	47.2 (3.7)	45.0 (2.3)	52.1 (3.0)	51.0 (3.1)	**0.015**
Waist circumference, cm	104.7 (0.7)	107.1 (1.0)	104.9 (1.2)	106.5 (1.0)	107.9 (1.3)	107.0 (1.1)	0.064
Hypertension, %	71.3 (2.1)	69.3 (1.9)	72.6 (2.5)	70.5 (1.8)	71.5 (3.6)	68.4 (3.4)	0.629
Systolic BP, mmHg	129.9 (0.8)	126.5 (1.0)	131.2 (1.2)	126.9 (0.9)	130.7 (1.5)	132.4 (1.4)	0.039*
Diastolic BP, mmHg	67.4 (0.9)	66.0 (0.9)	68.2 (0.9)	66.0 (1.2)	66.6 (0.8)	70.2 (1.0)	0.036*
Diabetes, %	33.0 (3.0)	31.0 (1.9)	31.4 (3.9)	31.4 (1.4)	31.3 (2.6)	37.6 (1.9)	0.217
Hemoglobin A1c, %	6.1 (0.1)	6.1 (0.0)	6.2 (0.1)	6.1 (0.1)	6.2 (0.1)	6.2 (0.0)	0.349
Dyslipidemia, %	55.9 (1.9)	53.2 (2.2)	66.3 (2.8)	64.7 (2.3)	66.2 (2.3)	64.4 (3.0)	** < 0.001**
Total cholesterol, mg/dL	185.5 (2.0)	180.9 (2.1)	182.5 (2.5)	176.4 (1.8)	176.4 (3.1)	174.4 (3.2)	**0.001**
HDL-C, mg/dL	49.5 (0.8)	49.3 (0.8)	48.8 (0.7)	51.2 (1.0)	51.3 (1.3)	50.0 (1.2)	0.227
Triglycerides, mg/dL	1.8 (0.1)	1.7 (0.1)	1.8 (0.1)	1.5 (0.1)	1.4 (0.1)	1.4 (0.1)	**0.002**

Bold fonts indicate statistically significant

*BMI*, body mass index; *BP*, blood pressure; *CVD*, cardiovascular disease; *HDL-C*, high-density lipoprotein-cholesterol; *IPR*, Income-to-poverty ratio; *NHANES*, national health and nutrition examination survey

*Tested for the non-linear trend

**Fig. 1:**
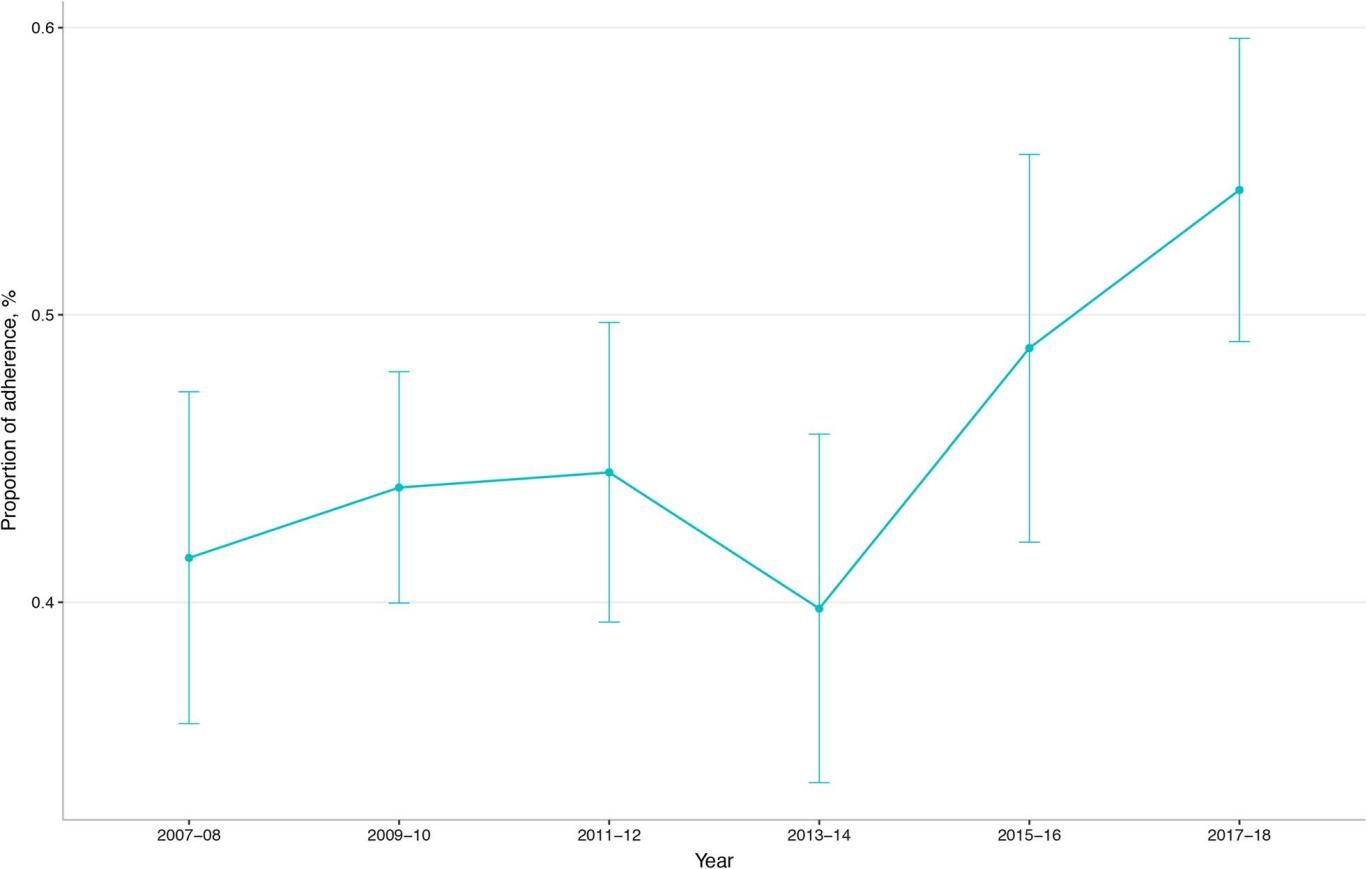
The crude weighted trend in adherence to the physical activity guideline for American among US adults with a history of cardiovascular disease, national health and nutrition examination survey 2007–08 to 2017–18

**Fig. 2:**
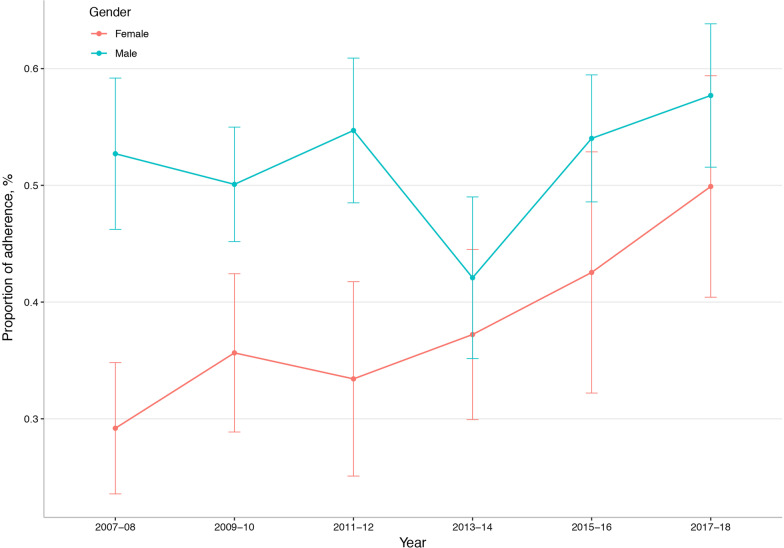
Crude weighted trends in adherence to the physical activity guideline for American among male and female US adults with a history of cardiovascular disease, national health and nutrition examination survey 2007–08 to 2017–18

The proportion of participants with a history of CVD adhering to the PAG in 2007–08 was 41.5% and it increased to 54.3% in 2017–18. There was a significant linear change in the trend (*P* for trend 0.002) (Fig. 1). The trend in adherence to the PAG was similar across different subgroups by age (*P* for interaction, 0.964), race/ethnicity (*P* for interaction, 0.571), education (*P* for interaction, 0.231), income (*P* for interaction, 0.103), and diagnosis (*P* for interaction, 0.915). Males had a higher proportion of adherence compared to females (*P* for interaction, 0.012), while the trend was only significant in females (*P* for trend < 0.001) (Fig. 2).

The association of adherence to the PAG with the cardiovascular risk factors among participants with a history of CVD was presented in Table 2. Among participants with a history of CVD, adherence to the PAG was significantly associated with a lower level of waist circumference (β-coefficient, − 3.6; 95% CI, − 5.2 to − 2.0), BMI (β-coefficient, − 1.3; 95% CI, − 2.0 to − 0.6); systolic BP (β-coefficient, − 4.2; 95% CI, − 6.1 to − 2.4), hemoglobin A1c (β-coefficient, − 2.0; 95% CI, − 0.4 to − 0.1), and triglycerides (β-coefficient, − 0.3; 95% CI, − 0.4 to − 0.1) and a higher level of diastolic BP (β-coefficient, 2.0; 95% CI, 0.8–3.2) in the regression model without adjustment of potential covariates. After adjustment of the covariates, the association remained significant between adherence to the PAG and the levels of waist circumference (β-coefficient, − 2.2; 95% CI, − 3.4 to − 1.0), BMI (β-coefficient, − 1.3; 95% CI, − 2.0 to − 0.5), hemoglobin A1c (β-coefficient, − 0.2; 95% CI, − 0.3 to − 0.1), and triglycerides (β-coefficient, − 0.2; 95% CI, − 0.4 to − 0.1).

**Table 2:** Differences in the cardiovascular risk factors among adults with a history of CVD who adhere or did not adhere to the

Risk factors	Adherence	Non-adherence	Beta coefficient 1#	Beta coefficient 2*
Waist circumference, cm	104.5 (103.4–105.6)	108.2 (107.0–109.4)	**−** **3.6 (−** **5.2 to **− **2.0)**	**−** **2.2 (−** **3.4 to −** **1.0)**
BMI	30.0 (29.5–30.4)	31.3 (30.8–31.8)	**−** **1.3 (−** **2.0 to −** **0.6)**	**−** **1.3 (−** **2.0 to −** **0.5)**
Systolic BP, mmHg	127.4 (125.8–129.0)	131.7 (130.6–132.7)	**−** **4.2 (−** **6.1 to −** **2.4)**	**−** 1.2 (**−** 3.2 to 0.8)
Diastolic BP, mmHg	68.5 (67.6–69.5)	66.6 (65.6–67.5)	**2.0 (0.8 to 3.2)**	0.2 (**−** 1.2 to 1.6)
Hemoglobin A1c, %	6.0 (6.0–6.1)	6.3 (6.2–6.4)	**−** **0.2 (−** **0.4 to −** **0.1)**	**−** **0.2 (−** **0.3 to −** **0.1)**
Total cholesterol, mg/dL	177.5 (174.2–180.7)	180.4 (178.0–182.8)	**−** 2.9 (**−** 7.0 to 1.1)	**−** 2.7 (**−** 7.3 to 1.9)
HDL-C, mg/dL	50.7 (49.5–52.0)	49.4 (48.5–50.4)	1.3 (**−** 0.2 to 2.8)	1.4 (**−** 0.2 to 3.0)
Triglycerides, mg/dL	1.4 (1.3–1.6)	1.7 (1.6–1.8)	**−** **0.3 (−** **0.4 to −** **0.1)**	**−** **0.2 (−** **0.4 to −** **0.1)**

Data are presented as mean with 95% confidence interval in parenthesis. Bold fonts indicate statistically significant

*BMI*, body mass index; *BP*, blood pressure, *CVD*, cardiovascular disease; *HDL-C*, high-density lipoprotein-cholesterol

#The regression model wasn’t adjusted for covariates. *The regression model was adjusted for age, gender, race/ethnicity, education, income, smoke, alcohol use, obesity, diabetes, hypertension, and dyslipidemia

## Discussion

Although the American Heart Association, the American College of Cardiology, and the American College of Sports Medicine have highlighted the importance of physical activity as a modifiable CVD risk factor, a sizable percentage of the US and worldwide population still present with low levels of physical activity because of various reasons: lack of time, physical limitation, personal barriers associated with perceived limitations in self-efficacy, and misconceptions of the volume of physical activity necessary for cardiovascular health benefits [11–13]. A major emphasis has been made on promoting exercise training and improving levels of cardiorespiratory fitness in the US and worldwide in an effort to prevent CVD [13, 14]. In this nationally representative study, we investigated the trends in adherence to the PAG for aerobic activity in US adults with a history of CVD. We found that the proportion of adherence to PAG significantly increased from 41.5% in 2007–08 to 54.3% in 2017–18, which increased by more than 30 percent. Moreover, this significant trend remained similar across different subgroups by age, race/ethnicity, education, income, and diagnosis. This welcome finding might be attributable to multiple factors, including the increasing awareness of the PAG and efforts to promote cardiovascular health [13–15].

The PAG for aerobic activity refers to all domains of aerobic activity, including leisure-time, work-related, and transportation-related aerobic activity. Previous studies have investigated the trends in adherence to the PAG in the US [5, 16–18]. However, most studies reported the trends based only on the leisure-time domain of aerobic physical activity [16–18], and these reported trends could not reflect the aerobic physical activity from work and transportation. In addition, we limited our participants to those with a history of CVD, among which the trend in adherence to the PAG has not been reported previously.

It is suggested that the adherence rates to the PAG varied with age, gender, race/ethnicity, education, and income [5, 18]. However, in the current study, we found that the trends were similar in different subgroups except for males and females. Similar to the previous report using objective measurements, we also found that females had lower physical activity levels [19]. Notwithstanding, it is delightful to find that the adherence rate to PAG in females had significantly increased from nearly 30% in 2007–08 to 50% in 2017–18.

For a long time, exercise training was strictly forbidden for patients with CVD such as chronic heart disease due to the fear that the increased load during exercise might harm the myocardium. This misunderstanding has been revised after several studies demonstrating the opposite effect [20, 21]. Actually, the cardioprotective effects of regular physical activity are clear and extend across all ages, sex, and race [22]. We evaluated the impact of adherence to the PAG on the cardiovascular risk factors among adults with a history of CVD, which has not been reported before. Unsurprisingly, adherence to the PAG was significantly associated with decreased levels of waist circumference, BMI, hemoglobin A1c, and triglycerides even after adjustment of the potential cofounders. Therefore, our study has important public health implications that the volume of physical activity recommended by the PAG is sufficient to provide cardiovascular health benefits for survivors with a history of CVD.

The strengths of the current study included using nationally representative data to allow the application of results to the entire US population. An additional strength is the robust quality assurance and control procedures during the process of data collection in NHANES, which improves the reliability of the data. However, our study also has several limitations. First, the history of CVD and information on physical activity were ascertained by self-report, which might be limited by misreporting and recall bias. The results of our study should therefore be interpreted with caution. Second, we were unable to evaluate the trends of muscle-strengthening activities in this study even though it was recommended by the PAG since the information about muscle-strengthening activity was not reported in NHANES 2007–18. Third, this is a cross-sectional study, no causal inference between adherence to the PAG and cardiovascular risk factors could be made, and the longitudinal relationship should be investigated by future prospective studies.

Based on the findings and limitations of the current study, there are some directions worth exploring in the future. The most important one and also the biggest challenge is how to increase adherence to the PAG since CVD survivors have reduced exercise capacity. Multidisciplinary efforts designed to increase adherence to PAG are warranted. Second, it is important to ascertain the type, duration and intensity of physical activities that are safe and most appropriate for CVD survivors since they are not able to exercise like the general population. Last, the effects of objectively measured physical activity on cardiovascular risk factors should be evaluated in future studies as physical activity estimates vary significantly depending on self-reported or measured objectively with accelerometers [8].

## Conclusion

In summary, this nationally representative study suggests that there is a significant increase in the proportion of adherence to the PAG for aerobic activity among US adults with a history of CVD from 2007–08 to 2017–18, and adherence to the PAG was associated with improvement in cardiovascular risk factors.

## Data Availability

The datasets generated and analyzed during the current study are available in the NHANES, https://www.cdc.gov/nchs/nhanes/index.htm

## Abbreviations

BP: Blood pressure
BMI: Body mass index
CI: Confidence interval
CVD: Cardiovascular disease
CV: Cardiovascular
NHANES: National health and nutrition examination survey
PAG: Physical activity guidelines
